# LIVING ALONE IS ASSOCIATED WITH POORER PHYSICAL FUNCTION AND BONE MINERAL DENSITY IN ICELANDIC OLD ADULTS

**DOI:** 10.1093/geroni/igz038.1793

**Published:** 2019-11-08

**Authors:** Alfons Ramel

**Affiliations:** University of Iceland, Reykjavik, Capital area, Iceland

## Abstract

Background: Loneliness and living alone have been significant public health concerns among older adults given their association with a wide range of adverse health outcomes. Aim: The aim of this study was to examine whether living alone is associated with physical function and bone health in community-dwelling older adults. Methods: This was a secondary analysis of existing cross-sectional data of old adults (N=182, 73.7±5.7yrs, 58.2% female) from the Reykjavik capital area in Iceland. Information on socioeconomics, health, dietary intake and physical function was collected. 25-hydroxy-vitamin D (25OHD) and bone mineral density (BM were grouped retrospectively into “living alone” and into “in cohabitation”. Results: Of our subjects, 76.4% were in cohabitation and and 23.6% lived alone. Participants who lived alone were older (74.5±5.6 vs. 72.1±5.0,P=0.008) and more often female (74.4 vs. 53.2%,P=0.014), but there were no differences in education, smoking, number of medications, physical activity (PA) or body mass index (BMI). According to age and gender corrected analyses, participants in cohabitation had higher grip strength (6.2±2.4lb,P=0.011), higher 25OHD (13.1±6.3nmol/L,P=0.037) and higher BMD (z-score lumbal: 1.195±0.417,P=0.005; z-score femur: 0.421±0.219,P=0.054; z-score total: 0.846±0.290,P=0.004). Statistical correction for PA, BMI, education and fish oil intake did not change the results. Conclusion: In comparison to old adults who live in cohabitation, Icelandic old adults who live alone have poorer physical function, lower 25OHD and lower BMD, which increases their risk for wrist or hip fracture. These differences between groups were not explained by physical, dietary or social confounding variables.

